# CLEC7A regulates M2 macrophages to suppress the immune microenvironment and implies poorer prognosis of glioma

**DOI:** 10.3389/fimmu.2024.1361351

**Published:** 2024-05-23

**Authors:** Jinchao Wang, Xiaoru Li, Kai Wang, Kaiji Li, Yalong Gao, Jianye Xu, Ruilong Peng, Xu Zhang, Shu Zhang, Yuan Zhou, Shangchen Xu, Jianning Zhang

**Affiliations:** ^1^ Key Laboratory of Post-Neuro Injury, Neuro-repair and Regeneration in Central Nervous System, Ministry of Education and Tianjin City, Tianjin Neurological Institute, Tianjin Medical University General Hospital, Tianjin Medical University, Tianjin, China; ^2^ Department of Neurosurgery, Shandong Provincial Hospital Affiliated to Shandong First Medical University, Shandong First Medical University, Jinan, China; ^3^ Department of Neurosurgery, Tianjin Huanhu Hospital, Tianjin, China; ^4^ School of Medicine, Nankai University, Tianjin, China; ^5^ Department of Neurosurgery, Tianjin Medical University General Hospital, Tianjin, China

**Keywords:** glioma, CLEC7A, macrophage, prognosis, tumor immunity

## Abstract

**Background:**

Gliomas constitute a category of malignant tumors originating from brain tissue, representing the majority of intracranial malignancies. Previous research has demonstrated the pivotal role of CLEC7A in the progression of various cancers, yet its specific implications within gliomas remain elusive. The primary objective of this study was to investigate the prognostic significance and immune therapeutic potential of CLEC7A in gliomas through the integration of bioinformatics and clinical pathological analyses.

**Methods:**

This investigation involved examining and validating the relationship between CLEC7A and glioma using samples from Hospital, along with data from TCGA, GEO, GTEx, and CGGA datasets. Subsequently, we explored its prognostic value, biological functions, expression location, and impact on immune cells within gliomas. Finally, we investigated its potential impact on the chemotaxis and polarization of macrophages.

**Results:**

The expression of CLEC7A is upregulated in gliomas, and its levels escalate with the malignancy of tumors, establishing it as an independent prognostic factor. Functional enrichment analysis revealed a significant correlation between CLEC7A and immune function. Subsequent examination of immune cell differential expression demonstrated a robust association between CLEC7A and M2 macrophages. This conclusion was further substantiated through single-cell analysis, immunofluorescence, and correlation studies. Finally, the knockout of CLEC7A in M2 macrophages resulted in a noteworthy reduction in macrophage chemotaxis and polarization factors.

**Conclusion:**

CLEC7A expression is intricately linked to the pathology and molecular characteristics of gliomas, establishing its role as an independent prognostic factor for gliomas and influencing macrophage function. It could be a promising target for immunotherapy in gliomas.

## Introduction

Gliomas represent the most frequently encountered primary tumors within the brain, constituting 81% of malignant neoplasms in the central nervous system (CNS). Despite the array of available treatment modalities, encompassing chemotherapy, radiotherapy, and surgery, the prognosis remains bleak. Previous studies data indicate that survival rates hinge on the pathological attributes of the tumor. Patients diagnosed with World Health Organization (WHO) grade I gliomas exhibit the most favorable relative survival rates, whereas those with WHO grade IV tumors display the lowest survival rates, with a mere 6.8% surviving beyond 5 years post-diagnosis (1). Consequently, there exists an urgent need to pinpoint innovative and highly efficacious treatment strategies for gliomas to enhance patient outcomes.

Immunotherapy has emerged as a promising treatment modality, enhancing the prognosis for patients with various cancers, including some central nervous system malignancies (2). Within the spectrum of immunotherapy approaches, the utilization of immune checkpoint blockade exhibits encouraging outcomes in patients with gliomas. This strategy aims to impede tumor progression by reactivating cytotoxic T cells directed against tumor cells. Despite gliomas being immunogenic tumors with elevated neoantigen levels, only a subset of patients responds to immune checkpoint blockade, potentially attributable to primary or acquired resistance (3). A distinctive feature of gliomas is the sparse infiltration of T cells, contrasting with the abundant infiltration of tumor-associated macrophages (TAMs) (4). The predominant TAM subtype in most malignancies is the M2-TAMs, exerting a pivotal role in immune suppression and treatment resistance (5, 6). Furthermore, a robust correlation is evident between the infiltration of M2-TAMs in the tumor microenvironment of high-grade glioma patients and diminished survival rates. The polarization of M2-TAMs in the tumor immunological microenvironment and their role in adapting to resist immune attacks constitute a crucial mechanism. Nevertheless, the process by which M2-TAMs polarize in gliomas, impeding anti-tumor immunity, remains elusive. Comprehending this intricate process and investigating potential therapeutic interventions to surmount immunotherapy resistance in gliomas represents a critical avenue for future research.

CLEC7A, also known as Dectin-1, is part of the C-type lectin superfamily, recognized as a subset of pattern recognition receptors (PRRs). Its role within the innate immune system is significant, particularly in identifying β-glucans, a type of polysaccharide present in the cell walls of fungi (7). This recognition initiates a signaling cascade that regulates various cellular responses, encompassing phagocytosis, autophagy, as well as the production of cytokines and chemokines (8). Recent investigations have unveiled potential connections between Dectin-1 and diverse biological processes such as immune homeostasis, autoimmunity, allergies, and cancer (9). Consequently, Dectin-1 emerges as a promising therapeutic target for managing both infectious and non-infectious diseases. Yet, the specific role of CLEC7A in gliomas and its impact on tumor immune responses remain inadequately elucidated.

This study employed bioinformatics and clinicopathological techniques to comprehensively evaluate the mRNA and protein expression levels of CLEC7A in gliomas. Subsequently, we assessed the prognostic significance of CLEC7A overexpression in glioma patients. Additionally, we investigated the potential mechanisms through which CLEC7A regulates the immunosuppressive microenvironment of gliomas. These findings suggest that CLEC7A could serve as an innovative prognostic marker and a promising therapeutic target for gliomas by combating M2 macrophages.

## Materials and methods

### Collection and organization of public data

We retrieved clinical and transcriptomic data for glioma patients from the TCGA, CGGA, GEO, and GTEx databases. In total, we amassed 693 samples from the CGGA database, 698 samples from the TCGA database, 63 samples from the GEO database (GSE50161), and 1152 samples from the GTEx database. To be included in our analysis, samples needed to meet specific criteria: 1) Computation of the mean for any duplicated patient sequencing outcomes within the database, and 2) Each selected sample had to include comprehensive clinical information about the patient, including survival time, survival status, age, and gender, among others. Samples lacking any of these crucial clinical details were systematically excluded from the study.

### CLEC7A expression analysis

In the initial phase, we conducted an analysis of CLEC7A expression in both gliomas and non-tumor brain tissues across the TCGA, GTEx, and GEO (GSE50161) databases. To delve deeper into the expression correlation between CLEC7A and molecular markers in glioma, we investigated the TCGA and CGGA databases, respectively. These pivotal molecular markers encompassed WHO grade (II, III, and IV), IDH mutation status (mutant or wildtype), 1p19q co-deletion status (codel or no-codel), and MGMT methylation status (methylated or unmethylated). The statistical analysis was executed using R software, and the presentation of results was facilitated by the “ggplot2” package.

### Prognostic analysis and establish nomograms prediction model

Survival outcomes among glioma patients were evaluated employing Kaplan-Meier analysis, incorporating data from diverse sources, including the TCGA and CGGA databases, as well as clinical cases from our hospital. The glioma cohort was stratified based on CLEC7A mRNA expression levels, leading to the identification of two distinct groups: the high expression group (50% to 100% expression) and the low expression group (0% to 50% expression). Statistical analysis and visualization were executed using the “survival” and “survminer” packages, respectively. To enhance clinicians’ ability to predict glioma patient prognosis, we performed a Nomograms analysis in the CGGA and TCGA databases. The Nomograms approach serves as a robust tool for evaluating an individual’s risk by integrating multiple factors in a clinical context. We identified independent risk factors for glioma prognosis and employed them to predict the likelihood of 1, 3, and 5-year overall survival.

### Patient selection

In this investigation, we enrolled 119 consecutive patients diagnosed with gliomas through pathological confirmation (**Table 1**). The cohort comprised individuals admitted to Shandong Provincial Hospital, affiliated with Shandong First Medical University, from February 2008 to February 2020. All participants exhibited a preoperative Karnofsky performance score exceeding 70. The pathological sections of these selected patients underwent re-evaluation and reclassification by two pathologists, adhering to the updated WHO classification (2021) criteria. Ethical clearance for this study was obtained from the Research Ethics Committee of Shandong Provincial Hospital, affiliated with Shandong First Medical University. The research commenced after obtaining written consent from all participants or their legal representatives.

**Table 1:** The clinical information corresponding to the clinical samples.

	Normal brain	Glioma			
		WHO II	WHO III	WHO IV	Total
Total no. of patients	6	40	32	47	119
Male	3	21	14	24	59
Female	3	19	18	23	60
Extent initial surgical resection (n)					
GTR		26	23	28	77
STR or biopsy		14	9	19	42
Radiotherapy					
Yes		30	22	36	88
No		10	10	11	31
Chemotherapy					
Yes		24	21	38	83
No		16	11	9	36
CLEC7A expression					
Low expression (TIS ≤ 4)	6	35	22	19	76
High expression (TIS > 4)	0	5	10	28	43
IDH-1/2 mutation					
Mutation		33	18	4	55
Wildtype		7	14	43	64

### Immunohistochemistry and immunofluorescence assessment

Our team has previously published a comprehensive protocol and scoring criteria for immunohistochemistry and immunofluorescence (10). In this study, the rabbit polyclonal anti-Dectin-1 antibody (Abcam, ab140039) was utilized, with dilutions of 5 µg/ml and 20 µg/ml. Two independent assessors, blinded to patient outcomes, conducted the semi-quantitative analysis.

### Functional enrichment analysis

The criteria for selecting differentially expressed genes (DEGs) in gliomas with high vs. low CLEC7AT expression involved choosing the top 500 genes with the highest coefficient of rank (COR) from both TCGA and CGGA datasets. The selected DEGs exhibited a P-value < 0.05. To explore potential underlying mechanisms, we performed Gene Ontology (GO) and Kyoto Encyclopedia of Genes and Genomes (KEGG) pathway analyses using DAVID 6.8 (https://david.abcc.ncifcrf.gov/). The study presents the top six results, ordered by ascending P-values (P < 0.05).

### Gene set variation analysis

The functional enrichment score for each glioma sample was computed utilizing the “GSVA” package with default settings. The gene set related to immune functions was acquired from GSEA (https://www.gsea-msigdb.org/gsea/index.jsp). The categorization of immune functions followed the guidelines outlined by GSEA. The enrichment results were visually represented through a heatmap generated with the “heatmap” package. Pearson correlation analysis was employed to assess the association between CLEC7A expression and multiple immune processes.

### Glioma immune microenvironment analysis

To scrutinize the association between CLEC7A and glioma immunity, we assessed immunity scores, stroma scores, ESTIMATE scores, and tumor purity in glioma patients exhibiting high or low CLEC7A expression in both the TCGA and CGGA databases. Employing the capabilities of CIBERSORT, our goal was to discern intricate connections between CLEC7A and the diverse landscape of immune cells infiltrating the glioma microenvironment. This comprehensive analysis played a pivotal role in unraveling potential immunomodulatory roles of CLEC7A in glioma pathogenesis. Additionally, our exploration into the correlation with immune checkpoint markers aimed to provide insights into the potential implications of CLEC7A in modulating immune responses within the context of glioma. These findings contribute significantly to a nuanced understanding of the molecular interplay in glioma, shedding light on potential avenues for therapeutic interventions. This research enhances our knowledge of the complex relationship between CLEC7A expression and glioma immunity, paving the way for further investigations and potential therapeutic strategies.

### Single-cell analysis of CLEC7A expression levels in glioma

Through the analysis of single-cell data, we can acquire more intricate information regarding gene alterations. The single-cell data were sourced from the CGGA and GEO databases (GSE70630, GSE84465, GSE89567). Utilizing the Seurat R software package, we reduced cell dimensions and generated a UMAP diagram to visualize cell types. This process involved the streamlining of cell dimensions and the creation of a UMAP diagram for visualizing distinct cell types, employing the Seurat R software package. To investigate the involvement of CLEC7A in tumors, we explored expression variations across a spectrum of cell types.

### Western blot analysis

Total protein was extracted following transfection of cells with siRNA. After BCA quantification, the proteins were denatured by boiling in 4x protein loading buffer, separated by sodium dodecyl sulfate–polyacrylamide gel electrophoresis (SDS-PAGE), and subsequently transferred to polyvinylidene difluoride membranes (0.45μm pore size, Millipore, Temecula, CA, USA). The membranes were then blocked and incubated overnight at 4°C with the following primary antibodies: CLEC7A (Abcam, ab217331, 1:500), CD163 (Abcam, ab182422, 1:1000), and GAPDH (Abclonal, A19056, 1:5000). The following day, membranes were washed three times for 10 minutes with TBST buffer and then incubated with species-appropriate horseradish peroxidase (HRP)-labeled secondary antibodies (1:5000, Cell Signaling Technology, USA) for 1 hour at room temperature. Finally, the immunoblot bands were visualized using an imaging system (Bio-Rad, Hercules, CA, USA) and quantified using ImageJ software (Version 1.46r, Wayne Raband, USA).

### Cell culture, macrophages polarization and siRNA transfection

RAW 264.7 cells were initially cultured in DMEM supplemented with 10% FBS. Subsequently, to induce polarization towards M1 macrophages, LPS (200ng/ml) was added to the culture medium, thereby exposing RAW 264.7 cells to pro-inflammatory stimulation. After 24 hours of incubation, IL-4 (10ng/ml) was further added to the medium to promote the transition of M1 macrophages towards the M2 phenotype. Finally, Western blot analysis was conducted to validate the phenotypic changes in the cells. M2 macrophages were transfected with non-silencing scrambled control siRNA (si-NC) and small interfering RNA (siRNA). The siRNAs were designed and synthesized by Hesheng Biotechnology Co., Ltd. (Beijing, China).

### Transwell assay

To assess whether the absence of CLEC7A impacts the chemotactic capability of macrophages, we conducted a transwell assay. In each well of the upper chamber, 200µl of suspension containing 5×10^5^ RAW 264.7 cells was added. Following polarization of M2 macrophages and siRNA transfection, GL261 mouse glioma cells were seeded into the lower chamber at a density of 1×10^6^ cells per well. The coculture of M2 macrophages and GL261 cells was maintained for 48 hours. The upper chamber was rinsed twice with PBS at 24, 48 hours, respectively. Subsequently, cells from the upper layer were removed using a wet swab. Cells that migrated to the bottom surface were fixed with 4% paraformaldehyde and stained with 0.5% crystal violet.

### Statistical analysis

A multiple group comparison was performed using Tukey’s test. For the comparison of continuous variables between two groups, the Wilcoxon rank-sum test was applied. To assess the linear relationship between two continuous variables, Pearson correlation analysis was conducted. All statistical analyses were performed using R software (version 4.1.3) and GraphPad prism 8. The prognostic value was estimated by univariate and multivariate Cox models using SPSS statistical software (version 25.0). All statistical tests were two sided, and a p-value < 0.05 was considered statistically significant in all analyses.

## Results

### The upregulation of CLEC7A expression is associated with the malignant phenotype of glioma

**Figure 1:**
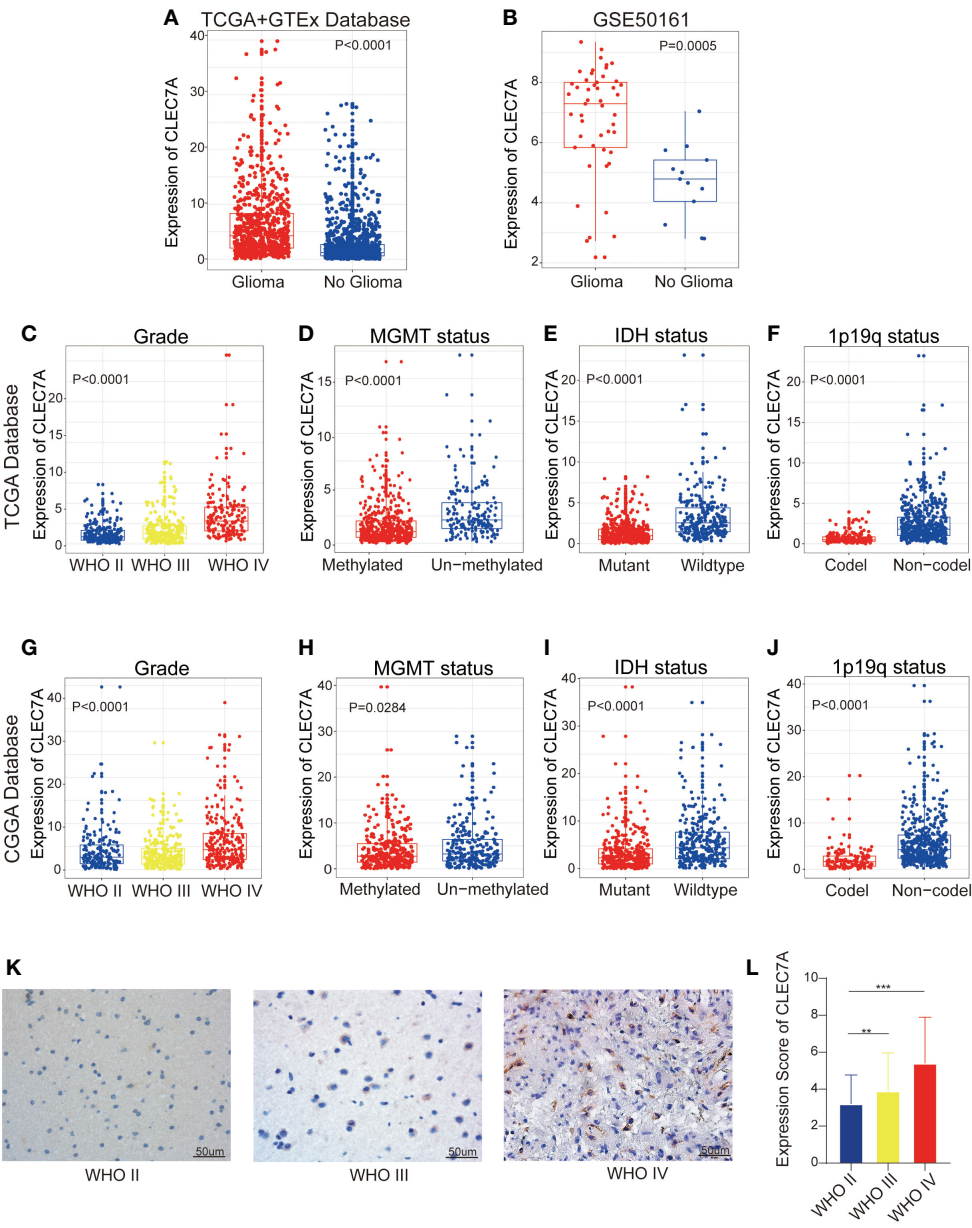
High expression of CLEC7A is associated with malignant glioma. CLEC7A was expressed in the TCGA database and the GTEx database was used as a control **(A)**. Comparison of CLEC7A expression in glioma patients and control patients in GEO database (GSE50161) **(B)**. CLEC7A expressions between distinct clinicopathological characteristics (WHO grade, IDH status, 1p/19q status, MGMT methylation status) in both the TCGA and CGGA dataset **(C-J)**. Representative CLEC7A expression in gliomas of different WHO grades by immunohistochemistry staining **(K)**. The immunohistochemical scores of CLEC7A were measured in different grades **(L)**. *p<0.05, **p<0.01, ***p<0.001.

To explore the involvement of CLEC7A in gliomas, we conducted an analysis of CLEC7A expression levels in both non-tumor brain tissue and glioma samples utilizing data from TCGA, GTEx, and GEO (GEO50161) dataset. The analysis unveiled a notable enrichment of CLEC7A expression in glioma samples (**Figures 1A, B**). Considering the molecular pathological heterogeneity of gliomas, we systematically scrutinized the RNA-seq data of glioma samples in accordance with WHO guidelines, performing a comparative expression analysis on samples from various groups. Within the TCGA database, CLEC7A demonstrated elevated expression in samples of high-grade gliomas, MGMT un-methylated status, IDH wildtype status, and those 1p/19q no-codel status, (**Figures 1C-F**). These findings were corroborated in the CGGA database (**Figures 1G-J**). Furthermore, we conducted immunohistochemical staining to validate the results from the database, utilizing clinical specimens we collected. Illustrative images derived from immunohistochemical staining are depicted in (**Figures 1K, L**). The findings disclosed a heightened expression of CLEC7A in glioma tissues, and its expression escalated with the tumor grade, aligning with the results in the database.

### CLEC7A is an independent prognostic factor in glioma patients

**Figure 2:**
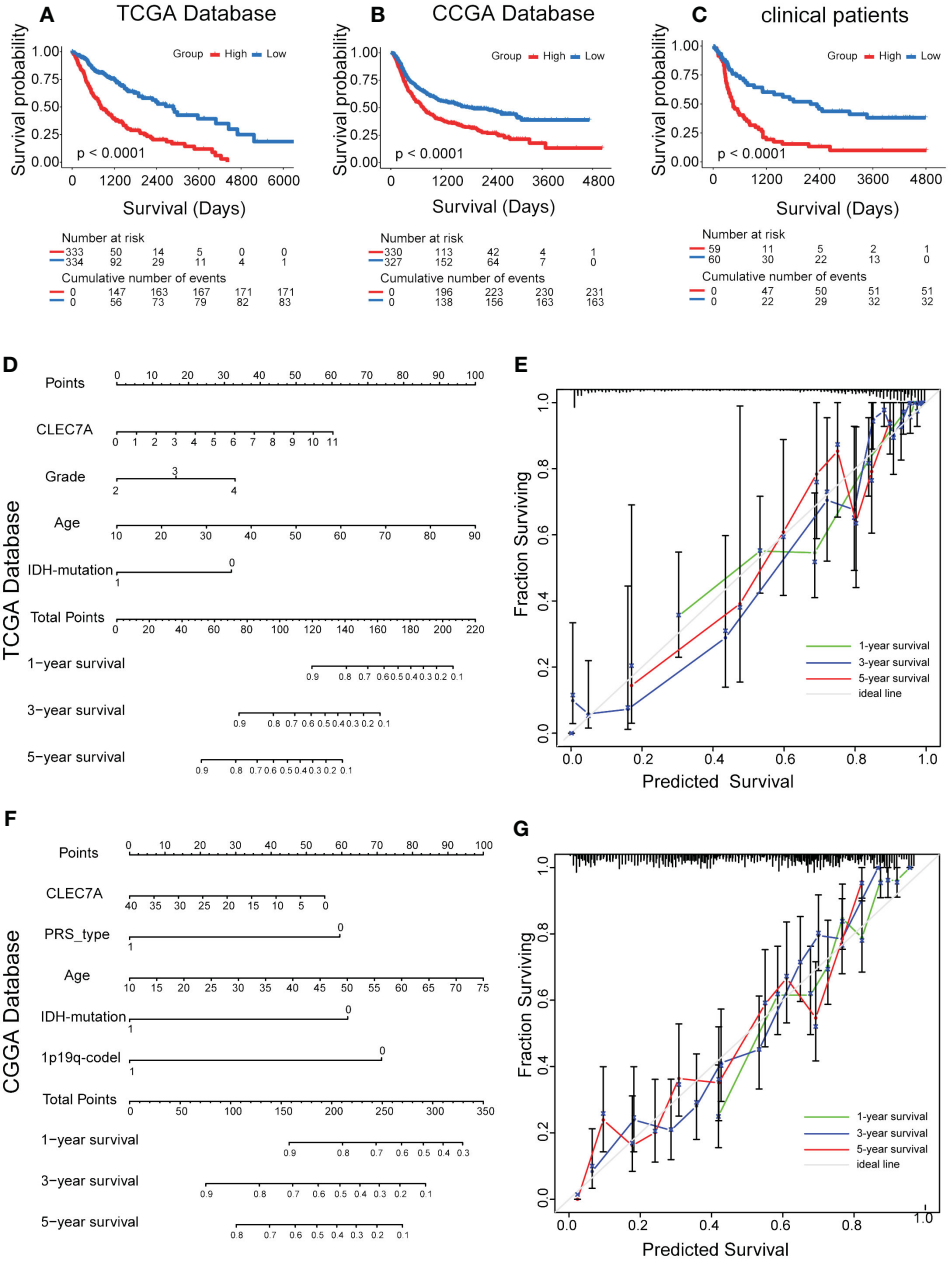
Effect of high CLEC7A protein expression on overall survival time in glioma patients. Kaplan-Meier analysis of CLEC7A expression in TCGA database **(A)**, CGGA database **(B)** and clinical samples **(C)**. Nomogram for the prediction of prognostic probabilities. The nomogram for the prediction of overall survival time was developed using the TCGA database **(D)** and CGGA database **(F)**. The calibration plots for predicting 1-, 3-, and 5-year survival using the TCGA database **(E)** and CGGA database **(G)**.

Our investigation delved deeper into the prognostic significance of CLEC7A in glioma. The findings revealed a noteworthy association between elevated CLEC7A expression and considerably poorer OS, as validated by Kaplan-Meier survival analysis utilizing both the TCGA and CGGA database (**Figures 2A, B**). Furthermore, to corroborate these results, we performed validation on patients from our hospital. The result established a significant correlation, demonstrating that high expression of CLEC7A was associated with reduced OS among glioma patients (**Figure 2C**).

Subsequently, we conducted both univariate and multivariate Cox regression analyses using data from the CCGA and TCGA database, revealing CLEC7A expression as an independent risk factor for gliomas (**Table 2**). This observation was further substantiated through validation in patients from our hospital (**Table 2**). In the Nomogram prediction model, each patient-specific characteristic corresponds to a different score. Patient 1, 3, and 5-year survival rates were predicted by summing the scores obtained for each glioma patient (**Figures 2D, F**). The agreement between actual and predicted survival rates was substantial, as evidenced by the calibration curves (**Figures 2E, G**).

**Table 2:** Univariate and multivariate analysis of prognostic parameters in CGGA database, TCGA database, and clinical samples overall survival.

	Univariate analysis			Multivariate analysis	
	HR (95%CL)	P-value		HR (95%CL)	P-value
TCGA database					
Age	1.072 (1.060-1.085)	9.18E-33***		1.039 (1.024-1.053)	6.14E-08***
Gender	1.002 (0.740-1.356)	0.991081		0.778 (0.569-1.064)	0.116085
Grade	5.285 (4.112-6.794)	1.32E-38***		2.218 (1.607-3.062)	1.29E-06***
IDH	10.342 (7.276-14.701)	9.30E-39***		2.230 (1.297-3.833)	0.003719**
1p/19q	4.314 (2.575-7.227)	2.79E-08***		1.589 (0.860-2.934)	0.138995
CLEC7A	1.181 (1.139-1.224)	1.04E-19***		1.065 (1.005-1.130)	0.033971*
CGGA database					
Age	1.026 (1.016-1.035)	6.84E-08***		1.008 (0.999-1.016)	0.067324
Gender	1.097 (0.882-1.363)	0.404903		0.994 (0.797-1.240)	0.959543
Grade	2.760 (2.353-3.236)	9.23E-36***		2.166 (1.814-2.587)	1.33E-17***
IDH	3.064 (2.459-3.818)	2.00E-23***		1.546 (1.187-2.014)	0.001241**
1p/19q	3.573 (2.546-5.016)	1.84E-13***		2.511 (1.733-3.639)	1.15E-06***
CLEC7A	1.041( 1.022-1.060)	1.93E-05***		0.978 (0.956-1.000)	0.048817*
Clinical database					
Age	1.029 (1.010-1.049)	0.003 **	1.013 (0.996-1.030)		0.146
Grade	2.811 (2.110-3.745)	<0.001 ***	2.489 (1.700-3.644)		<0.001 ***
Resection extent	2.939 (1.749-4.937)	<0.001 ***	3.284 (1.839-5.864)		<0.001 ***
Radiotherapy	0.767 (0.424-1.387)	0.380	0.773 (0.405-1.475)		0.435
Chemotherapy	1.693 (1.018-2.816)	0.042 *	0.561 (0.311-1.013)		0.055
IDH	4.609 (2.515-8.448)	<0.001 ***	1.629 (0.742-3.577)		0.224
CLEC7A	2.646 (1.678-4.171)	<0.001 ***	1.923 (1.192-3.103)		0.007 **

*p<0.05, **p<0.01, ***p<0.001.

### CLEC7A emerges as a promising biomarker for identifying the mesenchymal subtype in gliomas

**Figure 3:**
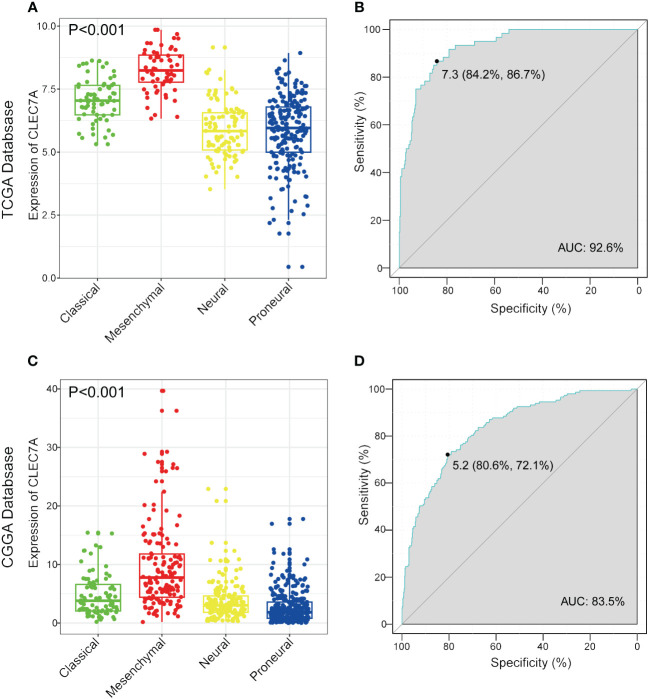
CLEC7A is a potential marker for malignant subtypes of gliomas. CLEC7A was highly expressed in the mesenchymal subtype in the TCGA database **(A)** and CGGA database **(C)**. ROC curve analysis showed that CLEC7A was highly sensitive and specific to predict the mesenchymal subtype in the TCGA database **(B)** and CGGA database **(D)**.

Transcriptional subtyping has gained widespread acceptance as a pivotal method for molecular classification in gliomas (11). In our examination of the molecular expression profile of CLEC7A, we conducted an analysis of its distribution across distinct molecular subtypes of glioma within the TCGA database. The results highlighted a substantial upregulation of CLEC7A in the mesenchymal subtype compared to the other three subtypes, evident in both the TCGA and CGGA datasets (**Figures 3A, C**). To robustly validate this observation, we utilized a receiver operating characteristic curve analysis for CLEC7A expression and its correlation with the mesenchymal subtype across gliomas of all grades. Notably, the area under the curve (AUC) for CLEC7A expression reached 92.6% and 83.5% for the TCGA and CGGA datasets, respectively (**Figures 3B, D**). These results strongly indicate an overexpression of CLEC7A in mesenchymal subtype gliomas, suggesting a potential oncogenic role in the progression of gliomas. Considering that mesenchymal glioblastomas are recognized for their particular aggressiveness, these findings substantiate our previous identification of the association of CLEC7A with high malignancy in brain tumors.

### CLEC7A is correlated with immune functions in glioma

**Figure 4:**
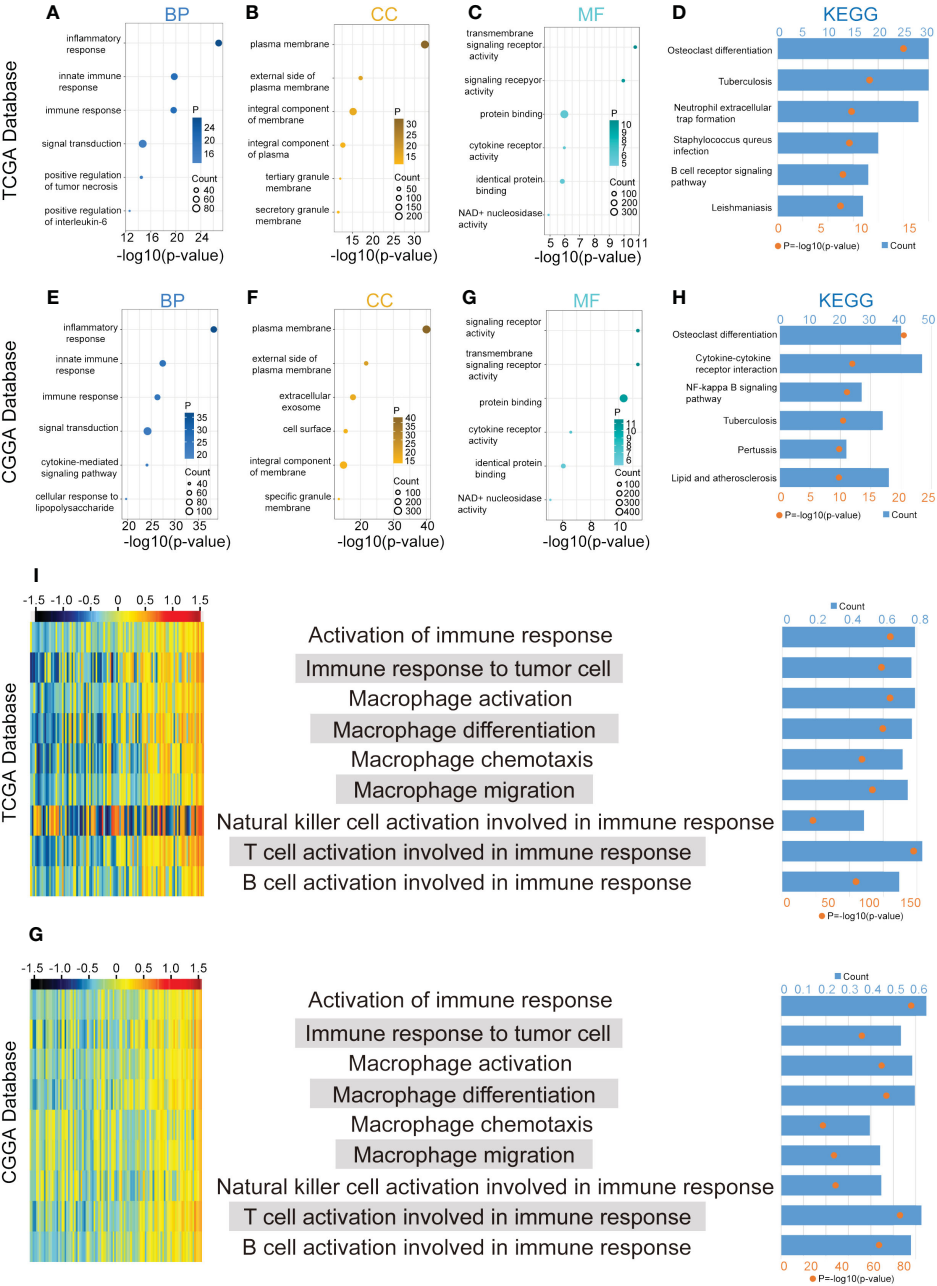
CLEC7A is closely associated with immune process regulation in gliomas. BP, CC, and MF are mostly related to CLEC7A in TCGA database **(A-C)** and CGGA database **(E-G)**. KEGG pathway analysis of CLEC7A in TCGA database **(D)** and CGGA database **(H)**. The heatmap showed the expression of CLEC7A and the enrichment scores of immune functions of each patient in the TCGA and CGGA database **(I, G)**. The samples were arranged in ascending order of the expression of CLEC7A. The column graph and line graph on the right showed the R-value and P-value of the correlation analysis.

GO and KEGG enrichment analyses provided valuable insights into the potential biological functions of CLEC7A based on DEG sets in both the TCGA and CGGA databases. In the TCGA database, the GO analysis revealed that CLEC7A is associated with several key biological processes (BP). Notably, it showed a significant involvement in the inflammatory response, immune response, and positive regulation of interleukin-6 production (**Figure 4A**). These findings suggest that CLEC7A may contribute to immune-related processes and inflammatory signaling pathways, which aligns with its potential role in glioma progression. Additionally, the most related cellular components (CC) to CLEC7A are located on the external side of the plasma membrane (**Figure 4B**). This information provides insights into the cellular localization of CLEC7A, which is crucial for understanding its interactions and potential functions in the context of gliomas. The molecular functions (MF) include transmembrane signaling receptor activity and protein binding (**Figure 4C**). This suggests that CLEC7A may function as a signaling receptor on the cell membrane and may be involved in protein-protein interactions, further implicating its role in cellular communication and signaling pathways. The CLEC7A-related signaling pathways identified are Osteoclast differentiation, Neutrophil extracellular trap formation, Staphylococcus aureus infection, B cell receptor signaling pathway (**Figure 4D**). These pathways are often linked to immune response and inflammatory processes, providing additional evidence supporting the involvement of CLEC7A in these critical biological functions. Similar results were obtained in the CGGA database, where the BP, CC, MF, and signaling pathways associated with CLEC7A mirrored those in the TCGA database (**Figures 4E-H**). This consistency across databases strengthens the reliability of the findings and further supports the notion that CLEC7A likely plays a crucial role in immune response and disease regulation.

### CLEC7A is positively associated with immune functions and inhibitory immune checkpoints

**Figure 5:**
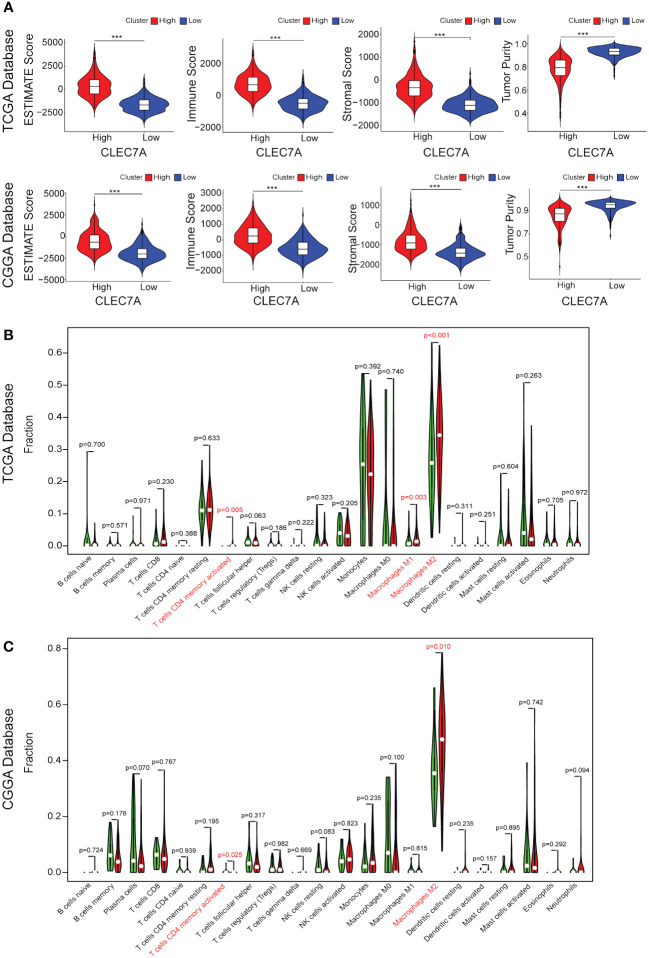
CLEC7A expression is associated with increased infiltration of immune cell. Correlation between CLEC7A expression and stromal score, immune score, tumor purity, or ESTIMATE score in the TCGA database and CGGA database **(A)**. Relationship between CLEC7A expression and presumed immune cell infiltration in glioma tissue in the TCGA database **(B)** and CGGA database **(C)**. ***p<0.001.

**Figure 6:**
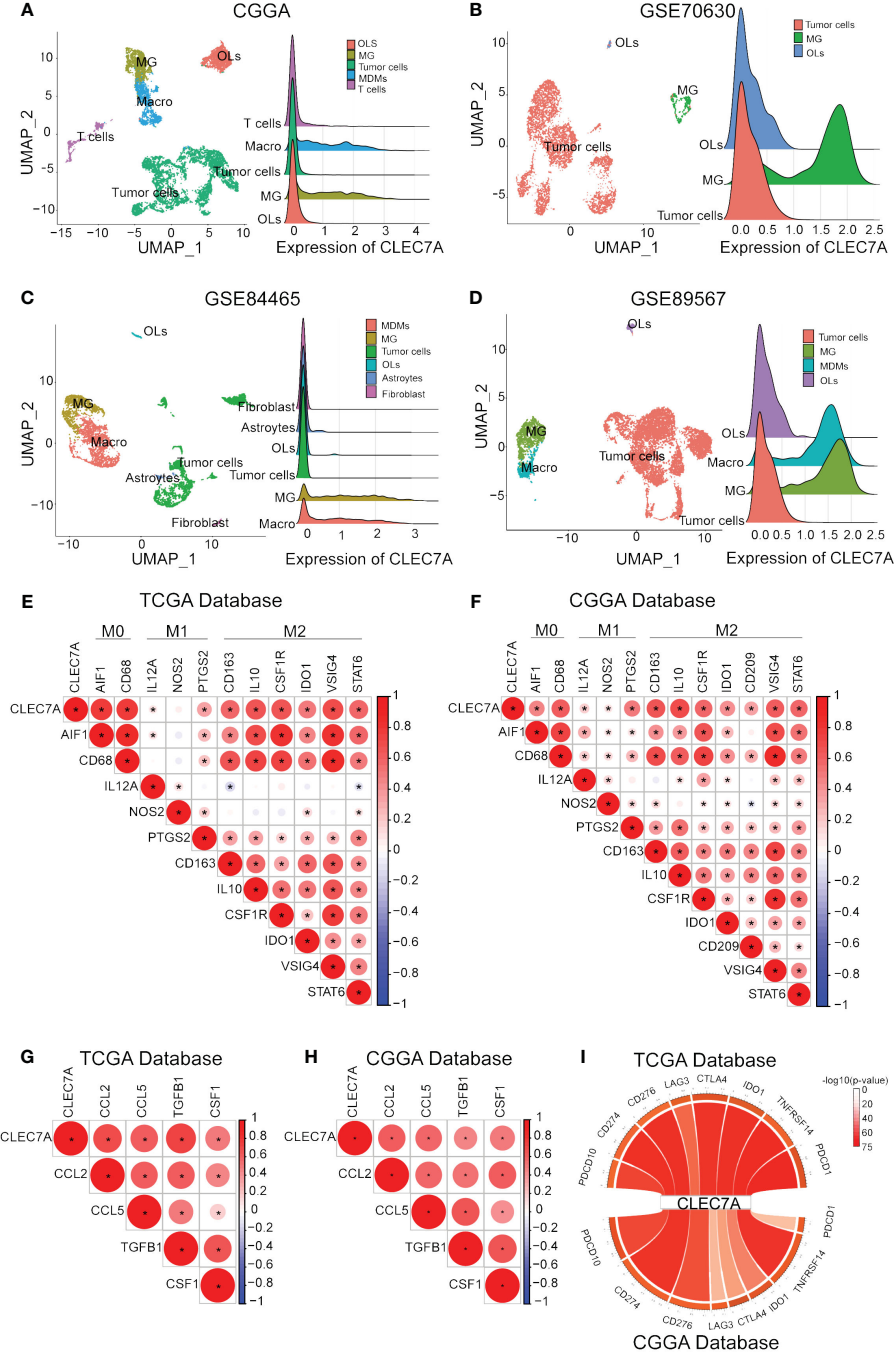
CLEC7A was positively correlated with macrophages and molecular markers of macrophage polarization and chemotaxis. The relationship between CLEC7A and different cells in CGGA, GSE70630, GSE89567, and GSE84465 databases. (OLs, oligodendrocyte; MG, Microglia; Macro, macrophages) **(A–D)**. Relationship between CLEC7A and important makes of macrophage subtypes in the TCGA database **(E)** and CGGA database **(F)**. Positive correlation between CLEC7A expression and molecular markers of macrophage polarization and chemotaxis in the TCGA database **(G)** and CGGA database **(H)**. CLEC7A expression was closely related to a variety of immune checkpoint genes, including PDCD10, CD274, PD-1, PD-2, CD276, LAG3, CTLA-4, IDO1, TNFRSF14, PDCD1 **(I)**.

To further validate the significance of CLEC7A in glioma immune reactions, we conducted GSVA to investigate its association with various immune processes. Utilizing data from the TCGA and CGGA databases, we assessed enrichment scores for immune processes. Our correlation analysis revealed a positive association between CLEC7A expression and several crucial immune processes (**Figures 4I, G**). Glioma patients with high CLEC7A expression exhibited elevated immune, stromal, ESTIMATE scores and lower tumor purity (**Figure 5A**). Furthermore, we examined the correlation between CLEC7A and established inhibitory immune checkpoints—such as PDCD10, CD274, PD-1, PD-2, CD276, LAG3, CTLA-4, IDO1, TNFRSF14, and PDCD1—in different databases (**Figure 6I**). CLEC7A demonstrated a strong positive correlation with these inhibitory immune checkpoints, which contribute to suppressing the immune response in gliomas. To summarize, our findings robustly support the involvement of CLEC7A in multiple aspects of immune responses within the glioma microenvironment.

### CLEC7A is closely related to macrophages

During the course of tumor immunity, a diverse array of immune cells, comprising T cells, NK cells, macrophages, and others, are mobilized to instigate immune responses with the objective of eradicating cancerous cells (12). To further the immune cells most associated with CLEC7A in glioma tissues, we applied CIBERSORT to classify and analyze the proportion of 22 immune cells retrieved from the TCGA and CGGA databases. Examination of TCGA-derived samples uncovered a notable increase in the proportion of CD4 memory-activated T cells, M1 macrophages, and M2 macrophages within the CLEC7A high-expression cohort of glioma patients (**Figure 5B**). Similar findings were replicated in the CGGA database, demonstrating significantly elevated proportions of CD4 memory-activated T cells and M2 macrophages (**Figure 5C**). To validate the association between CLEC7A expression and macrophages and microglia in gliomas, we analyzed single-cell data from the CGGAS database and the GEO database (GSE70630, GSE84465, GSE89567). The results unequivocally demonstrate that within glioma tissues, CLEC7A is predominantly expressed on tumor-associated macrophages. (**Figures 6A-D**). Consequently, we can infer a strong correlation between tumor-associated macrophages and CLEC7A expression within the glioma immune microenvironment.

### CLEC7A is associated with M2 macrophages

**Figure 7:**
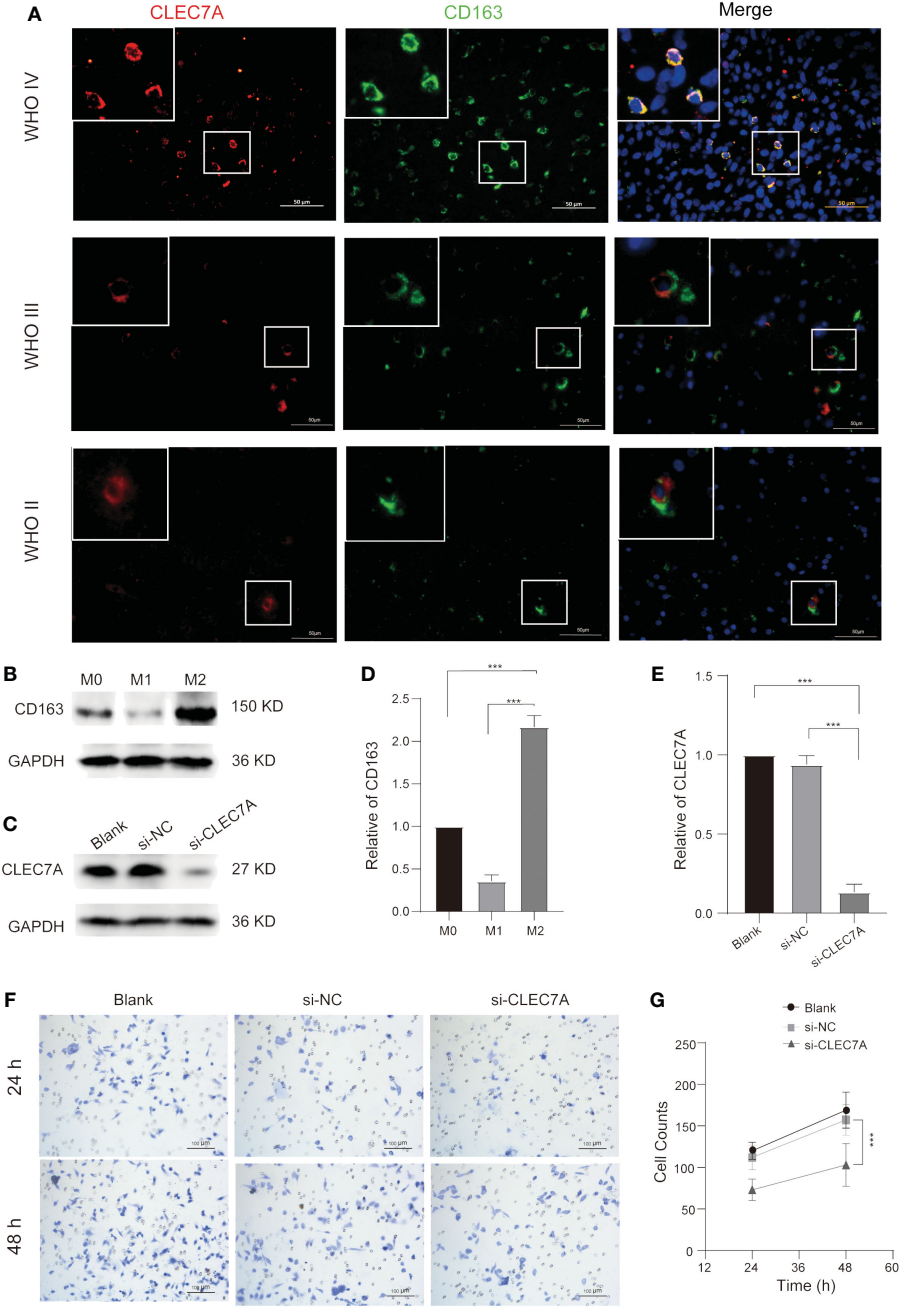
CLEC7A co-localizes with M2 macrophages and influences M2 macrophage chemotactic function. Immunofluorescence shows that CLEC7A colocalizes with CD163 in different grades of gliomas **(A)**. The expression of CD163 and its statistical analysis at different stages during the process of macrophage polarization **(B, D)**. The expression of CLEC7A and its statistical analysis in the blank group, si-NC, and si-CLEC7A groups **(C, E)**. Representative images of the Transwell assay in the blank group, si-NC, and si-CLEC7A groups **(F)**. The number of cells in the si-NC group was significantly higher than that in the si-CLEC7A groups **(G)**. *** P < 0.001.

Our findings above confirm that CLEC7A is closely associated with macrophages. We integrated data from diverse databases to explore the correlation between CLEC7A and macrophage polarization markers, including AIF1, CD68, IL12A, NOS2, PTGS2, CD163, IL10, CSF1R, IDO1, VSIG4, and STAT6. The analysis shows that there is a significant similarity between the results in the TCGA and CGGA databases (**Figures 6E, F**). The results showed that CLEC7A was found to have a more significant correlation with the markers of M2 macrophages. To verify if CLEC7A is expressed on M2 macrophages, we used immunofluorescence to assess the co-localization of CLEC7A with the established M2 macrophage marker CD163. The results unequivocally revealed a substantial co-localization of CLEC7A with CD163 (**Figure 7A**). In addition, a significant increase in CLEC7A+ CD163+ cells was observed with increasing WHO grade of glioma. In conclusion, our findings strongly suggest that CLEC7A is highly expressed in M2 macrophages in glioma tissues.

### Predicting the effect of CLEC7A on macrophages in gliomas

To elucidate the specific impact of CLEC7A on macrophages, we conducted correlation analyses to explore the relationship between CLEC7A and genes associated with glioma chemotaxis and polarization in publicly available databases. Our investigation revealed a positive correlation between the expression of CLEC7A and monocyte chemoattractant factors (CCL2, CCL5) as well as factors implicated in M2 macrophage polarization (TGF-β1, CSF-1) in glioma samples obtained from both TCGA and CGGA databases (**Figures 6G, H**). To confirm these findings, we employed si-RNA to knockdown CLEC7A in M2 macrophages (**Figures 7C, E**). Meanwhile, in order to ascertain phenotypic changes during macrophage polarization, we utilized Western blot analysis to determine the expression of CD163 (**Figures 7B, D**). The effectiveness of the knockdown was assessed using Western blot analysis. Subsequently, a Transwell assay was conducted to demonstrate the impact of CLEC7A on the chemotaxis of M2 macrophages towards glioma cells (**Figures 7F, G**). These findings support our hypothesis that CLEC7A may play a role in the chemotaxis and polarization of macrophages in gliomas.

## Discussion

Glioma remains the most invasive tumor within the CNS despite numerous efforts to reduce mortality (13). Although immunotherapy has shown efficacy in various cancers, its clinical impact on glioma patients remains suboptimal. This challenge stems from the distinctive inhibitory immune microenvironment in gliomas, commonly referred to as immunological cold disease (14). Due to the immunological characteristics unique to gliomas, the T cell content is relatively low, while macrophage content is high. This phenomenon underscores the potential for utilizing macrophages in the treatment of gliomas. Innovative approaches for immune-targeted cancer treatment could concentrate on selectively disrupting M2-TAM signaling cascades or inducing a polarization shift from a tumor-promoting M2-TAM phenotype to a tumor-killing M1 phenotype. The heightened infiltration of TAMs is correlated with a reduced long-term prognosis, as observed in both preclinical models and clinical investigations (7, 15). It is widely acknowledged that TAMs play a pivotal role in the progression of most tumors, and M2-TAM specifically promote glioma development (16, 17).

The protein dectin-1, encoded by CLEC7A, emerges as a promising candidate for therapeutic intervention. Its potential therapeutic significance has been documented in urothelial bladder cancer and intestinal tumors (18, 19). To investigate the relationship between CLEC7A and gliomas, we first performed expression and survival analysis based on data from different databases and clinical patient information. The results showed that CLEC7A expression is increased in gliomas, and its expression levels are correlated with WHO grades. Patients with high CLEC7A expression have worse prognosis, and CLEC7A is an independent prognostic factor for gliomas. The findings from enrichment analysis suggest that CLEC7A may be associated with immune infiltration in gliomas. To ascertain the immune cell most significantly correlated with CLEC7A, we employed CIBERSORT analysis and single-cell analysis and found that macrophages exhibited the most notable difference between high and low CLEC7A expression levels. To further explore the relationship between CLEC7A and macrophage subtypes, we conducted correlation analysis between CLEC7A and markers representing different subtypes. The results indicate that CLEC7A is most closely associated with M2 macrophages. Our clinical patient immunofluorescence results also support this conclusion. To predict the impact of CLEC7A on M2 macrophages in gliomas, we knocked out CLEC7A in M2 macrophages. The results showed that macrophage chemotaxis was significantly reduced. Therefore, we posit that CLEC7A may play a role in the processes of macrophage chemotaxis and polarization in gliomas.

Moreover, CLEC7A exhibits a significant elevation in mesenchymal subtype gliomas, which are distinguished by heightened immunosuppression, increased aggressiveness, and malignant proliferation driven by mesenchymal differentiation resulting from mutations in the NF1 gene (11). Previous investigations have demonstrated an upregulation of immune checkpoint gene expression in this particular tumor subtype in comparison to the remaining three subtypes (20). Consequently, CLEC7A potentially contributes to the clinical progression of gliomas by influencing the immune system. These findings underscore the involvement of CLEC7A in the malignant mechanisms of gliomas. Therefore, a comprehensive exploration of the mechanistic role of CLEC7A in gliomas could pave the way for the identification of novel therapeutic interventions to counteract this formidable malignancy.

Gliomas themselves have immunosuppressive properties that hinder the body’s immune surveillance against the tumor. Previous studies have demonstrated that immune checkpoint molecules including PD-L1, CTLA-4 and IDO are associated with immune evasion in glioma cells (21, 22). Immune checkpoint inhibitors unlock the suppression of the immune system and reactivate the killing effect of immune cells, such as T-cells, on tumor cells (23). Although favorable treatment effects have been observed in preclinical glioma studies, the clinical efficacy of these inhibitors has not been satisfactory (23). In our study, we analyzed data from various databases and observed a positive correlation between CLEC7A and that of several important immune checkpoint genes that have been previously characterized. Given its immunomodulatory function, we hypothesize that CLEC7A may be a potential immune checkpoint in gliomas. It could be an adjunctive therapy to the existing imperfect immune checkpoint inhibitors.

PRRs are pivotal components of innate immunity, playing a critical role in eliciting immediate responses to pathogens and laying the groundwork for adaptive immunity (24). Activation of PRRs not only induces the production of cytokines but enhances phagocytic, microbicidal, and antigen-presentation capabilities. Within the spectrum of membrane-bound PRRs, C-type lectin receptors (CLRs) constitute a substantial group dedicated to recognizing extracellular pathogens, sharing a common feature of a carbohydrate-binding C-type lectin domain (9). Among the well-explored CLRs, Dectin-1 stands out as a prominent example, specifically designed to recognize β-glucan structures that are notably prevalent in fungal cell walls (25). Simply put, PRR like Dectin-1 serve as one of the first lines of immune defense, able to sense abnormal cells at a low threshold and activate immune cells for enhanced response. In our study, we predicted that Dectin-1 promotes macrophage polarization. The finding that Dectin-1 promotes macrophage polarization is supported by studies in myocardial ischemia/reperfusion injury (26) and melanoma (27). This creates an intriguing situation in gliomas, where molecules that initially recognize tumor cells and can attract macrophages instead contribute to the potential polarization of macrophages into tumor-promoting M2-TAM. This may be one of the mechanisms of immune escape from glioma cells.

While our study has uncovered significant insights, it’s crucial to acknowledge limitations. The sample size for immunohistochemical staining and survival analysis was small, necessitating caution in result interpretation. The role of CLEC7A in glioma-induced immunosuppression is inferred from bioinformatics analysis, highlighting the need for functional experiments to understand its dysregulation fully. Conducting these experiments is vital to assess the potential efficacy of CLEC7A inhibitors in preclinical glioma studies, offering a foundation for innovative immunotherapeutic strategies to enhance patient prognosis.

## Data availability statement

The original contributions presented in the study are included in the article/supplementary material. Further inquiries can be directed to the corresponding authors.

## Ethics statement

The studies involving human participants were reviewed and approved by Research Ethics Committee of Shandong Provincial Hospital Affiliated to Shandong First Medical University. The patients/participants provided their written informed consent to participate in this study.

## Author contributions

JW: Formal analysis, Writing – original draft, Writing – review & editing. KW: Investigation, Software, Writing – original draft, Writing – review & editing, Validation. XL: Supervision, Validation, Writing – original draft, Writing – review & editing. KL: Data curation, Validation, Writing – original draft, Writing – review & editing. YG: Resources, Visualization, Writing – original draft, Writing – review & editing. JX: Methodology, Project administration, Writing – original draft, Writing – review & editing. RP: Project administration, Software, Writing – original draft, Writing – review & editing. XZ: Data curation, Methodology, Writing – original draft, Writing – review & editing. SX: Data curation, Resources, Writing – original draft, Writing – review & editing. SZ: Writing – original draft, Writing – review & editing. JZ: Funding acquisition, Methodology, Writing – original draft, Writing – review & editing. YZ: Methodology, Writing - review & editing.
